# Pulsed Electric Field Treatment of Sweet Potatoes to Reduce Oil and Acrylamide in Kettle Chips

**DOI:** 10.3390/foods14040577

**Published:** 2025-02-10

**Authors:** Mark M. Skinner, Morgan A. Fong, Tauras P. Rimkus, Alyssa N. Hendricks, Tina P. Truong, Luke G. Woodbury, Xinzhu Pu, Owen M. McDougal

**Affiliations:** 1 Micron School of Materials Science and Engineering, Boise State University, Boise, ID 83725, USA; markskinner@u.boisestate.edu; 2 Department of Chemistry and Biochemistry, Boise State University, Boise, ID 83725, USA; morganfong@u.boisestate.edu (M.A.F.); taurasrimkus@u.boisestate.edu (T.P.R.); alyssahendricks@u.boisestate.edu (A.N.H.); 3 Food and Dairy Innovation Center, Boise State University, Boise, ID 83725, USA; tinatruong@boisestate.edu; 4 Biomolecular Sciences Ph.D. Program, Boise State University, Boise, ID 83725, USA; lukewoodbury@boisestate.edu; 5 Biomedical Research Institute, Boise State University, Boise, ID 83725, USA; shinpu@boisestate.edu

**Keywords:** pulsed electric field, kettle chips, triple quadrupole mass spectrometry, oil retention, acrylamide production

## Abstract

The purpose of this investigation was to utilize pulsed electric field (PEF) technology to make sweet potato kettle chips (SPKC) healthier by lowering the amount of oil absorbed and reducing the amount of acrylamide formed during frying. Sweet potatoes were treated continuously in an Elea PEF Advantage Belt One system and prepared as SPKC, without peeling and sliced to a thickness of 1.7 mm. The specific energy for PEF application was set to either low (1.5 kJ/kg) or high (3.0 kJ/kg) with a field strength of 1.0 kV/cm and a pulse width of 6 μm. Batches of 500 g unrinsed potato slices were fried in canola oil at 130 °C for 360 s. The oil content in 3.0 g of fried SPKC was 1.39 g or 46.3%, whereas the oil content was 37.9% for high and 37.7% for low PEF-treatment conditions. Acrylamide (AA) in the fried SPKC was quantified by mass spectrometry to be 0.668 μg/g in the non-PEF control and 0.498 μg/g for low and 0.370 μg/g for high PEF treatment. The results of this study support the use of PEF in SPKC processing to reduce oil absorbance during frying by up to 9% and lower AA by up to 45%.

## 1. Introduction

Potato chips cater to diverse consumer tastes, making them popular snack foods in the United States (US) and across the world [1]. Traditional potato chip processing includes rinsing, removing the skin, thin slicing, and continuous conveyed frying of the potato slices in cooking oil [2]. The frying process includes the loss of water from the potato and the uptake of oil. When the potato slices reach a moisture content of just under 2%, the chip has generally achieved a desired golden-brown color and crispy texture [2]. Industrially processed chipping potato varieties are often selected based on low quantities of reducing sugars—glucose and fructose [3]. White-fleshed potato varieties used to make potato chips are round–oblong, smooth-skinned, and shallow-eyed [4]. In the US, Lamoka potatoes are the most common chipper variety because their low reducing sugar content minimizes the formation of undesirable brown colors and bitter flavors during frying [5]. As a point of reference, Russet variety potatoes, commonly used for French fries, hash browns, tater tots, and baked potatoes, generally have on the order of 1.6 mg/g and 2.9 mg/g of glucose and fructose, respectively, in the raw tuber [6]. By comparison, a Lamoka variety of potatoes contains on the order of 1.2 mg/g and 0.61 mg/g of glucose and fructose, respectively [7]. 

Lamoka potato chips are sliced to a thickness of around 1.4 mm before being fried, via a continuous process, in hot oil of 175 °C for about three minutes [7]. A recent study of continuous-style potato chips, made from Lamoka potatoes, demonstrated that pulsed electric field (PEF) treatment, followed by slicing and soaking, could lower the levels of reducing sugars (i.e., glucose and fructose) by 19% and asparagine (Asn) by 42%. The PEF-treated chips could be cooked in oil at a lower temperature (i.e., from 150 °C to 170 °C), conditions that reduced oil absorbance by 8% and provided a finished product with 29% less acrylamide (AA). In Santiago et al., 2024, the PEF specific energy and field strength for continuous style potato chips that provided optimal results for oil and AA reduction were 1.0 kV/cm for field strength and a specific energy of 1.5 kJ/kg [7]. Based on this precedent, a field strength of 1.0 kV/cm was maintained, and experiments were planned for specific energies of 1.5 kJ/kg and 3.0 kJ/kg for the thicker kettle chips. It stands to reason that sweet potato kettle chips (SPKC) may experience changes in oil retention, AA production, and AA precursors (i.e., Asn and reducing sugars) as a result of PEF treatment, similar to the observations of Santiago et al., 2024 [7].

Kettle chips are a popular snack food, available in most US grocery stores. Unlike continuous-style potato chips, kettle-style chips are not rinsed post-slicing, and the surface starch adds extra crispness and crunchiness to the final product. Kettle chips are thicker than continuous-style chips, and they require a lower frying temperature of about 130 °C and a longer fry time of six minutes to mitigate excessive production of melanoidins [8]. Kettle chips are approximately 0.3 mm thicker than continuous-style potato chips, i.e., 1.5 mm to 1.7 mm [9,10]. In Bernard’s 1985 patent and Renshaw et al., 2016, kettle chip thicknesses of 1.8 mm and 1.7 mm, respectively, were used as the basis for their studies [9,10]. Based on literature precedent and consultation with a local kettle chip processor, the current study used a chip thickness of 1.7 mm for all experiments. The result of a longer fry time and thicker composition is that fried kettle chips have higher total oil content, upwards of 48% vs. 28% for continuous fry, and higher moisture content, i.e., 5% vs. 2.5% to 3%, respectively [11].

Potato chips that brown too fast can appear burnt and low in quality, and thus will be used for less profitable purposes such as animal feed [12]. The brown color of the fried chip is attributed to melanoidins produced by the Maillard reaction, which occurs when reducing sugars react with amino acids, often Asn, during frying [13]. Melanoidins are responsible for the consumer-desired light brown coloration and favorable aroma of cooked foods [14]. When fried foods brown too fast, they will not reach low enough moisture content at the desired color. The optimal moisture content for chips is dependent on slice thickness and affects the shelf stability of the final product as it relates to crispness and oxidative degradation of oil [15].

**Figure 1:**
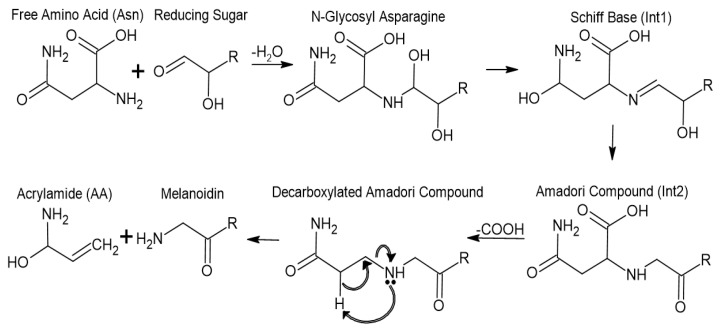
The mechanism of formation for AA and melanoidin by the Maillard reaction from Asn and glucose, derived from Parker et al., 2012 [13].

In Figure 1, AA is produced when amino acids and reducing sugars interact with water. The compounds undergo dehydration, which leads to a Schiff base or intermediate 1 (int1). From int1, an Amadori compound or intermediate 2 (int2) is formed, followed by decarboxylation and the formation of AA and a melanoidin compound.

The Maillard reaction occurs when foods containing amino acids like Asn, reducing sugars, and water are cooked at temperatures that exceed 120 °C; these conditions are common for French fries and other fried potato products [16]. The Maillard reaction is responsible for the formation of the probable carcinogen AA and melanoidins (see Figure 1). Some methods to mitigate AA formation in foods have used hot water (i.e., blanching) or chemicals to disrupt or minimize the production of AA. Vinci et al. (2012) provide a review of more than 30 papers that describe chemical interactions in the Maillard reaction with compounds intended to reduce AA formation, such as citric acid and asparaginase [17]. AA is believed to cause cancer and reproductive toxicity [18]. Due to the presence of AA in potato chips, California Proposition 65 requires that a warning label be present on the package if levels are higher than 250 ng/g, and the European Commission has imposed a limit of 750 ng/g of AA for food products [19,20,21].

Covington sweet potatoes are a robust cultivar that have smooth copper skin with deep orange flesh [22]. They have a lower glycemic index by comparison to traditional, white-fleshed chipping potatoes such as Atlantic or Lamoka [11]. Sweet potatoes have become a popular variety of tuber for kettle chips because of their high levels of vitamin A, vitamin B_6_, vitamin C, vitamin E, and dietary fiber [23]. The starch in sweet potato products has a lower glycemic index that can be tolerated by individuals with diabetic conditions [23,24].

In recent studies reported by Liu et al. (2023 and 2024), sweet potato chip analysis was performed following PEF treatment [25,26]. The focus of this current experimental study was to replicate methods and standards used in the US to produce SPKC [25,26]. Whereas Liu et al. (2023 and 2024) studied the Ao Red sweet potato, the current investigation used Covington sweet potatoes that were grown in California, USA [25,26]. In Liu et al. (2023 and 2024, sweet potato chips were cut by hand with a knife to a thickness of 3 mm, which was nearly twice that of typical 1.7 mm kettle chips made in the US [25,26]. In Liu et al. (2023 and 2024), raw tubers were sliced prior to singular PEF treatment in a lab-made chamber [25,26]. Liu et al. (2023 and 2024) utilized additional steps of blanching and ultrasound application to lower reducing sugar and Asn [25,26]. The sweet potato chips in Liu et al., 2023 and 2024 experiments were fried at a steady temperature of 150 °C, which was not the same batch method used for kettle chip frying in the US that begins at 130 °C for 120 s, before the heating element is reenergized [25,26].

Two issues with SPKC are high oil content and elevated AA production. Oil retention is problematic because it can lead to accelerated degradation of food quality as evidenced by off odor, poor taste, and soft texture for kettle-style chips [27]. High oil content also correlates with higher calorie levels.

A technological solution to mitigate oil absorption and reduce AA formation in potato chip processing is pulsed electrical field (PEF) technology. PEF application has been used to treat potatoes before they are sliced and fried so that they are easier to slice, have a smoother cut surface, cook faster, absorb less oil, and produce less AA [28,29]. PEF systems deliver repetitive electrical pulses of voltage over a short period of time (e.g., nanoseconds or milliseconds) [30]. The PEF-generated electric field produces pores in the cell walls of the potato, a phenomenon referred to as electroporation [31,32]. It has been demonstrated that PEF treatment of potatoes facilitates removal of reducing sugars and Asn from the tuber flesh, lowering the formation of AA during frying. The electroporation of potato cells has also been postulated to reduce oil absorbance due to the formation of an outer barrier on the surface of the chip caused by accelerated effusion of water and other small molecules during frying [29,33,34,35]. PEF treatment softens the potato tissue, resulting in cleaner slices with smoother surfaces that reduce oil entrapment on the surface of the potato during frying [29,34,35].

While many studies have been conducted on the use of PEF for French fries and continuous-style potato chips, few have been reported for kettle chips. The purpose of the current investigation was to determine the effect of PEF treatment to lower oil content (Theorem 1) and AA formation (Theorem 2) in SPKC; see the Material and Methods Section for theorems. We hypothesized that the amount of oil absorbed during the batch frying of PEF-treated SPKC would be reduced, and the amount of AA in the final product would be lower. PEF treatment to electroporate cell membranes has been postulated to provide egress pathways for AA precursors to effuse out of the potato flesh during frying [28,36,37]. In the case of French fries, the final fried product has on the order of 45–50% moisture, whereas a SPKC is below 5%. PEF treatment opens cell membranes to allow more water to leave the potato flesh during frying. The steam barrier formed around the frying potato is expected to be more resistant to oil absorbance during frying for the thicker and lower surface area French fry as compared to the SPKC. Thus, we expect PEF to lower oil content for SPKCs, but not to the degree that it is reduced for French fries. The basis for PEF to reduce both oil absorbance and acrylamide formation in fried potato products has been well established for French fries, providing a scientifically sound rationale for practical adoption in SPKC processing; the extent of the PEF effect is the focus of the current study.

## 2. Materials and Methods

### 2.1. Sample Preparations

#### 2.1.1. Sweet Potato Product

California-grown Covington sweet potatoes (*Ipomoea batatas*) were purchased in bulk (i.e., 50 lb. box) from a local grocery store in Boise, ID, USA, in September of 2023, rinsed, PEF-treated, sliced, fried, and stored.

#### 2.1.2. PEF Treatment

A PEF Advantage™ Belt One (ELEA, Quakenbrück, Germany) system was used to treat the whole raw tuber sweet potatoes prior to slicing. The conveyor belt was set to 0.14 m/s to pass the potatoes through a saline water bath with a salinity of 800 μS/cm, where PEF application occurred between electrodes separated by a distance of 24 cm. The specific energy for PEF treatment was set to either 1.5 kJ/kg or 3.0 kJ/kg, at a fixed electric field strength of 1 kV/cm, frequency of 20 Hz, and pulse width of 6 μs. The control group for this experiment included sweet potatoes that were identically processed but not treated with PEF.

#### 2.1.3. Slicing

The non-PEF control and PEF-treated sweet potato samples were sliced to a verified thickness of 1.7 mm using a Model CC slicer (Urschel Laboratories, Chesterton, IL, USA) and then separated into 500 g batches.

#### 2.1.4. Frying

An 11 kW deep-fat fryer (ELEA, Germany) filled with canola oil to the 20.9 L capacity (Signature Select, Boise, ID, USA) was used to conduct frying experiments. SPKC was fried for 360 s at an initial oil temperature of 130 °C. Upon submersion of the SPKC into the fryer oil, the power supply to the heating element was switched off, and the sweet potato slices were allowed to fry for 120 s, at which time the heating coil was reenergized. During the initial 120 s frying phase, the oil temperature dropped between 15 and 25 °C. Fryer temperature was measured with a centrally located internal thermocouple and an external thermocouple.

#### 2.1.5. Post Frying

**Figure 2:**
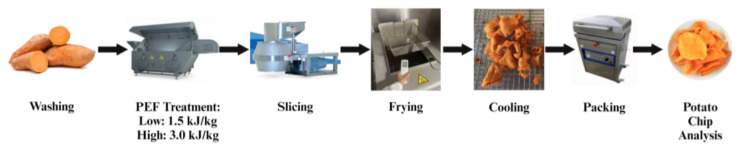
Sweet potato kettle chips were prepared by rinsing the tubers, PEF treating, slicing to 1.7 mm thickness, frying at 130 °C, cooling, and packing in a nitrogen environment until they were analyzed. Created with BioRender.com URL (accessed on 16 July 2024) [38].

Upon completion of frying, at a total time of 360 s, the SPKC were removed from the fryer oil, placed on a rack to drain, and cooled to room temperature. The fried SPKC were stored in UV-protective Mylar bags under nitrogen. A representation of kettle chip processing steps is shown in Figure 2.

### 2.2. Colorimetry

The SPKC (100 g) was placed onto a sample dish at room temperature and pressed firmly using a compression pan. The compression pan was removed. This method ensured a uniform height and opacity of these samples. Color quantification of the SPKC samples was measured using an Aeros D25 NC colorimeter (Hunterlab, Reston, VA, USA), equipped with a full spectrum LED 400–700 nm light source. The reflectance of light from the samples was measured with the spectrophotometer at five measurements per second and 25 measurements per rotational cycle. CIE tristimulus color values were collected and categorized using L* a* b* scale values.

### 2.3. Sweet Potato Kettle Chip Flour Preparation

The fried SPKC were frozen in liquid nitrogen and ground to a fine-grained flour using a CB15 commercial blender (Waring Commercial, Stamford, CT, USA). The SPKC flour was evaluated for oil content, AA, Asn, and reducing sugars.

### 2.4. Oil Content

The Signature Select canola oil used to fry the SPKC was extracted from the potato flour with diethyl ether (Fisher Chemical, Pittsburgh, PA, USA) using a solvent Auto Extractor, SER158 (VELP Scientifica, Usmate, Italy). The determination of crude fat in the fried potatoes was determined in accordance with AOAC method 963.15 [39]. The prepared SPKC flour (3 g) along with anhydrous sodium sulfate (Fisher Scientific, Pittsburgh, PA, USA, 2 g) was placed directly into 33 × 80 mm Soxhlet extraction thimbles (Fisher Brand, Achern-Fautenbach, Germany). The thimbles were placed into Randall glass extraction vessels, and diethyl ether (100 mL) was added. Following reflux, the Randall flasks were placed in the drying oven at 105 °C for one hour to remove residual ether. Oil percentage was calculated from the mass difference of the empty initial dry flask from the final flask with residual oil.

### 2.5. Acrylamide Quantification by Liquid Chromatography–Tandem Mass Spectrometry (LC-MS/MS)

#### 2.5.1. Acrylamide Extraction

Ground SPKC flour, of 1.0 g sample size, was placed into 50.0 mL centrifuge tubes, and the Agilent QuEChERS extraction method was used [40]. Samples in the centrifuge tubes were spiked with 500 µL of ^13^C_3_ AA (1,2,3-^13^C_3_, 99%, Cambridge Isotopes Laboratories) internal standard. High-performance liquid chromatography (HPLC) grade hexanes (5 mL, Millipore Corporation, Burlington, MA, USA) were added to the sample and vortexed in a Digital Vortex Mixer (DVM) 945,415 (Fisher Scientific, USA) for 10.0 s to separate oils. Deionized water (10.0 mL, NanoPure, Warszawa, Poland) and Optima LC/MS grade acetonitrile (10.0 mL, Fisher Chemical) were added to the solution with magnesium sulfate (4.0 g, Thermo Scientific, Waltham, MA, USA), sodium chloride (1.0 g, Thermo Scientific), trisodium citrate (1.0 g, Thermo Scientific), and disodium citrate (0.5 g, Thermo Scientific). The sample was vortexed on the DVM for 60 s and then centrifuged in a model 5920R (Eppendorf Laboratory Equipment, Hamburg, Germany) at 5000 rpm at 20 °C for 5 min. An aliquot of 1.0 mL was taken from the middle acetonitrile layer and added to a 2 mL centrifuge tube along with magnesium sulfate (150 mg, Thermo Scientific) and primary secondary amine sorbent (50.0 mg, Thermo Scientific). The sample was vortexed in the DVM for 30 s. Each sample was centrifuged at 6030 rpm for 60 s. Aliquots of 500 µL of this solution were filtered using a 0.45 µm cellulose centrifugal filter at 8090 rpm for 10 min in a microcentrifuge (EKF Diagnostics, Boerne, TX, USA) and analyzed by LC-MS/MS.

#### 2.5.2. LC-MS/MS Quantification of AA

The liquid chromatography separation of analytes was performed using an Agilent 1290 Infinity II system (Agilent, Santa Clara, CA, USA). Five microliters of sample were injected into a Hypercarb column (100 mm × 3 mm, 3 µL, Thermo Scientific, Waltham, MA, USA). LC elution mobile phases consisted of A (0.1% formic acid in water) and B (0.1% formic acid in methanol). The elution method was isocratic at 10% B for 6 min. Column was then washed with 90% B for 2.5 min and equilibrated with 10% B for 5 min before the next injection. The LC flow rate was maintained at 300 µL/min. Multiple reaction monitoring (MRM) was performed in positive ionization mode on an Agilent 6470 triple quadrupole mass spectrometer equipped with an electrospray source (Table 1).

### 2.6. Acrylamide Precursor Quantification

The precursors to AA are Asn and the reducing sugars glucose and fructose. The amounts of these precursors were quantified as described below.

#### 2.6.1. Asparagine Quantification

SPKC flour (1.5 g) was placed in 50 mL centrifuge tubes within a solution of 1% glacial acetic acid (99%, Sigma Aldrich, St. Louis, MO, USA) in acetonitrile (15 mL, Fisher Chemical) and nanopure water (15 mL). The QuEChERS extraction method was used to prepare each sample [40]. The samples were vortexed in the DVM for 1 min and then centrifuged for 5 min at 4000 rpm. The lower aqueous layer was extracted from the tube and filtered into 2 mL amber autosampler vials with Titan 3, 0.45 µm, PTFE membrane filters (Thermo Fisher, Waltham, MA, USA) and stored at 4 °C for liquid chromatography—mass spectrometry (LC-MS) analysis in accordance with the method by Henderson et al., 2010 [41]. Samples were manually injected into sample vials with micropipettes, increasing all reagents, diluents, and samples tenfold. Samples were analyzed using a four-point standard curve for both L-asparagine (Neogen Megazyme, Bray, Ireland).

#### 2.6.2. Reducing Sugar Quantification

Ground sweet potato sample (0.5 g) was added to 20 mL of DI water in a 50 mL Eppendorf tube following the protocol for the Luna Omega Sugar product and application guide [42]. The mixture was sonicated at 50 °C for 30 min in a Fisherbrand™ Digital Heated Ultrasonic Cleaner (Thermo Fisher, USA). Subsequently, chloroform (20 mL, Thermo Fisher) was added to the same Eppendorf tube, and the mixture was sonicated for an additional 15 min at the same temperature. After sonication, the samples were allowed to cool to room temperature. The top layer was decanted into another Eppendorf tube, and the solution was centrifuged for 10 min at 5000 rpm. The solution on top of the settled solid layer was then filtered through Titan 3, 0.45 µm, PTFE membrane filters (Thermo Fisher, USA) and analyzed in triplicate using an UltiMate 3000 HPLC (Thermo Fisher, USA) with a Luna Omega 3 μm SUGAR column and a Security Guard HPLC Guard Cartridge System (Phenomenex, Torrence, CA, USA). The injection volume was set at 10 μL, and the column temperature was 35 °C. The two mobile phases used were run using an isocratic method at 25% nanopure water and 75% acetonitrile (Fisher Chemical, USA). After separation, target particles were analyzed using a 5081.0020 Corona Veo RS (Thermo Scientific, USA) charged aerosol detector (CAD). Samples were analyzed using a four-point standard curve for both d-glucose (Sigma Aldrich, Wuxi City, China, >99.5%) and d-fructose (Tokyo Chemical Industry, Tokyo, Japan, >99.0%).

### 2.7. Statistical Analyses

Data are presented as means ± standard deviations for all analyses. All data were analyzed via analysis of variance Dunnett’s test using JMP Pro (v.18.0, SAS Institute Inc., Cary, NC, USA). Differences among different treatments were assessed with significance at *p* < 0.05.

## 3. Results

Table 2 represents the percentage of oil retained and the AA concentrations in μg/g or parts per million (ppm) for the finished, fried SPKC product. These values correlate with respect to the specific energy applied to the whole unsliced and uncooked tuber. The samples for oil and AA were analyzed in triplicate (n = 3), and mean values are listed below with standard deviation, where the letter n represents the number of replicates.

Table 3 lists the concentration of Asn, fructose, and glucose in μg/g (ppm) for the finished, fried SPKC product. These values correlate with respect to the specific energy applied to the whole unsliced and uncooked tuber. Duplicate samples were analyzed for Asn. The samples for reducing sugars were analyzed in triplicate, and mean values are listed below with standard deviation (see Table 3).

Table 4 lists the color values using the L*a*b* scale of the SPKC, i.e., the parameters signified L* (lightness 100 to darkness 0), a* (+red to −green), and b* (+yellow to −blue). Colorimetry for kettle chips was measured in triplicate and the mean value was listed below followed by standard deviation values in Table 4. Significant values in the low PEF-treated SPKC indicate a significant change in brightness, redness, and yellowness.

**Theorem** **1.** *The use of a pulsed electric field on SPKC reduces fryer oil retention in the final product.*

**Proof** **of Theorem 1.** Using continuous Randall extraction on 3.0 g aliquots of finished SPKC, the mean change in oil by weight was quantified and extrapolated. For a 3.0 g fried SPKC sample, PEF reduced the oil content from the non-PEF control of 1.39 g to the low PEF treatment of 1.14 g and the high PEF treatment of 1.13 g. On average, this was an 8.4% and 8.6% reduction in oil retained by weight for low and high PEF treatment, respectively (Table 2). The *p*-value for the low and high PEF settings was significant at 0.0049 and 0.0055. □

**Theorem** **2.** *The use of a pulsed electric field on SPKC reduces the production of AA in the final product.*

**Proof** **of Theorem 2.** The average concentration of AA produced during the 360 s frying time of finished SPKC was quantified and calculated using LC-MS/MS. It was determined that the use of PEF technology reduced the amount of AA measured in quadruplicate from 0.668 μg/g in the control to 0.498 μg/g (low PEF) and 0.370 μg/g (high PEF) (Table 2). Compared to the control sample, the *p*-value for low PEF was insignificant at 0.067, and for high PEF it was significant at 0.003. PEF application at 3 kJ/kg decreased AA concentration in the SPKC by 44.6% compared to the untreated control. □

## 4. Discussion

### 4.1. Oil Analysis

Canola oil was chosen in this experiment because major kettle chip manufacturers such as Kettle Brand^®^ Chips use it in kettle chip production [43]. It was experimentally determined that SPKC treated at the low and high level PEF treatments retained 8.4% and 8.6% less oil, respectively, from the fryer than non-PEF treated SPKC, equivalent to an average of 250 mg/g less oil. It is suspected that the combination of vapor barrier and surface smoothing effect, induced by PEF treatment, reduced entrapment of oil, contributing to the lower oil content in the fried SPKC [29,34,35]. Lower oil content will promote longer shelf life for products, since stored chips that are high in polyunsaturated oils are more prone to product degradation [44]. Reduced oil levels will proportionally reduce the overall caloric value of the final food product since canola oil contains 8.84 calories per gram [45]. According to the US Food and Drug Administration, the serving size is 13 kettle chips at around 28 g [46]. For non-PEF treated chips, the extrapolated values would be 114 calories, and a PEF treated sample would be 94 calories per serving. On a large industrial scale, e.g., during the production of frying foods, the lower uptake of oil will cumulatively mitigate oil overuse and waste by 2.5 mg/g of overall weight. The retail price range for US canola oil is between USD 0.87 and USD 1.27 per kg [47]. The application of PEF prior to frying will save food producers money by leaving more oil in the fryer and 2.5 mg/g less oil in the processed food product [3]. If a kettle chip company processes 226,000 kg (500,000 lbs) in a day, that would conserve approximately 567 kg of oil per day [48].

### 4.2. Acrylamide

The average amount of AA that is formed in non-PEF treated control SPKC is 668 ppm (Table 2). The intent of this experiment was to identify optimal PEF treatment conditions to lower the production of AA in SPKC, similar to what was observed for continuous style Lamoka potato chips [7]. However, in this experiment, each sample was fried on a kettle chip curve initiated at 130 °C for 360 s. It was the finding of this study that, with the same frying method and Covington sweet potato variety, a significant reduction in AA directly correlated with the PEF treatment intensity. PEF induces electroporation of cell membranes in the potato flesh. For the Maillard reaction to occur, Asn must come in contact with reducing sugars and water during frying. When kettle chips are not rinsed after PEF treatment, the reducing sugar content outside of the cells increases. During frying, we suspect the vapor barrier and caramelization of reducing sugars on the outside of the chip do not provide favorable conditions for the Maillard reaction. Literature precedent supports PEF-induced electroporation (Xu et al., 2020), and our results measured higher reducing sugars but lower AA [29]. Thus, we conclude that PEF does more to affect the mechanism of AA formation than can be inferred from the increased sugar content and consistent Asn content (Table 3). Based on the mechanism provided in Figure 1, the water available for the Maillard reaction to proceed is the most likely reason for the observed AA reduction when PEF is used. The *p*-values calculated from Dunnett’s tests verified the resulting difference in concentrations of AA with respect to PEF treatment was only significant for the high PEF at 3.0 kJ/kg treatment when compared to the non-PEF treated control. The high PEF treatment resulted in a significant reduction of 44.6% in AA compared to the untreated control. In Liu et al. (2024), the percent AA reduction was 85.9% due to the implementation of ultrasound and a PEF-specific energy of 9.47 kJ/kg [26]. The mean AA concentrations for fried SPKCs were 0.668 μg/g (control), 0.498 μg/g (low PEF), and 0.370 μg/g (high PEF), which are below the European Commission’s AA limit of 0.750 μg/g for food products, but above the California Proposition 65 level of 0.250 μg/g, necessitating a warning label for the chip package. The results indicate that specific energies above 3.0 kJ/kg may be used to achieve a higher reduction in AA concentration compared to untreated SPKC.

#### 4.2.1. Free Amino Acids—Asparagine

The free amino acid analysis for Asn (n = 2) shows a nonsignificant trend in Asn from the control concentration of 0.545 ppm to the samples treated at low and high specific energies (see Table 3). The absence of rinsing the SPKC after PEF treatment and slicing appears to have retained Asn at slightly higher levels in the PEF-treated cases. This result, where the PEF-treated samples had a higher concentration of Asn by comparison to the untreated control, was contrary to other experiments where the chips were rinsed or soaked prior to frying [36,49].

#### 4.2.2. Reducing Sugars

The concentrations of reducing sugar levels in samples for fructose and glucose with no PEF treatment and PEF treatment (i.e., low and high PEF) are listed in Table 3. Since SPKC were not rinsed after slicing, reducing sugars remained with the SPKC as they were placed in the fryer [50]. The *p*-values extrapolated during Dunnett’s testing verified that there was only a significant increase in glucose concentration at the high PEF level treatment, indicating that higher specific energies above 3.0 kJ/kg could potentially achieve higher concentrations of reducing sugars and more efficient frying.

### 4.3. Color Data

The sensory data available for PEF-treated SPKCs in the current study were limited to colorimetry. From the colorimetry data, a significant change was noticed in the low PEF setting for all values of L* brightness, a* redness, and b* yellowness. In the case of high PEF-treated SPKC, only the a* redness value increased significantly. The calorimetry data indicate that despite the higher concentration of reducing sugars for PEF-treated SPKC samples, the fried chips were lighter in color. This finding is interesting because reducing sugar content is higher, chip color is lighter, and AA levels are lower.

## 5. Conclusions

The values shown in Table 2 constitute a statistically significant reduction in retained fryer oil by about 8.5% for both high and low PEF settings. Compared to the control, AA levels for PEF-treated samples at low and high PEF were reduced by 0.298 μg/g and 0.170 μg/g, respectively. The 8.5% reduction in oil content and 45% lower AA in the SPKC, at the high PEF treatment level of 3.0 kJ/kg, were statistically significant when analyzed with Dunnett’s testing. The results of this study support the adoption of PEF in commercial kettle chip processing to mitigate oil absorption and AA production in order to reduce oil cost and consumption of AA. A single kettle chip processing facility, based on Kettle Brand^®^ chips, processes on the order of 227,000 kg (500,000 lbs) of kettle chips per day. Adoption of PEF technology can lead to a reduction of 567 kg of oil per day, i.e., 8.5% measured in the current study. A 55 gal. drum of canola oil has a market value of USD 770 USD and a mass of approximately 200 kg. Each day a SPKC processing facility would be expected to save USD 2183, which equates to approximately USD 800,000 per year in oil savings alone. The study by Liu et al. (2023) treated sweet potatoes with PEF at a specific energy of 9.47 kJ/kg and a field strength of 1.0 kV/cm, resulting in a reduction of oil absorbed by 31.9% and AA formed by 85.9% [25]. During the course of the current investigation, SPKC prepared at a thickness of 1.7 mm retained desirable texture and crispiness at PEF specific energy levels up to and including 3.0 kJ/kg. Based on these data, higher PEF levels reduce oil and acrylamide in fried SPKC, but excessive PEF specific energy will degrade product quality. This research has the potential to benefit many foods that are cooked with reducing sugars and free amino acids, i.e., vegetable chips.

## Figures and Tables

**Table 1:** MRM settings for the analysis of AA and acrylamide-D3 using an Agilent 6470 triple quadrupole mass spectrometer.

Analyte	Quantification		Confirmation		Fragmentor (V)	Cell Accelerator (V)
	Transition (*m*/*z*)	Collision Energy (V)	Transition (*m*/*z*)	Collision Energy (V)		
**Acrylamide**	72 > 55	12	72 > 27	28	50	4
**Acrylamide-D3**	75 > 58	12	75 > 30	32	40	4

**Table 2:** All quantified and calculated mean values of oil and AA are listed, followed by standard deviation based on sampling in triplicate (n = 3).

Specific Energy (kJ/kg)	Oil Retention (%)	Acrylamide (μg/g)
**Control**	46.3 ± 4.0	0.668 ± 0.100
**1.5 (low PEF)**	37.9 * ± 1.0	0.498 ± 0.050
**3.0 (high PEF)**	37.7 * ± 4.0	0.370 * ± 0.100

* Represents Dunnett’s testing values that are significant (*p* < 0.05).

**Table 3:** All quantified and calculated mean values of Asn (n = 2) and reducing sugars (n = 3). Standard deviation is provided for reducing sugars following the mean value in the table, but not Asn due to duplicate sampling.

Specific Energy (kJ/kg)	Asn (μg/g)	Fructose (μg/g)	Glucose (μg/g)
**Control**	0.545	0.354 ± 0.030	0.027 ± 0.004
**1.5 (low PEF)**	0.591	0.380 ± 0.020	0.041 ± 0.002
**3.0 (high PEF)**	0.586	0.437 ± 0.040	0.051 * ± 0.008

* Represents Dunnett’s testing values that are significant (*p* < 0.05).

**Table 4:** The colorimetry data mean values followed by standard deviation values. The number of replicates (n = 3).

Color	L*	a*	b*
**Control**	42.9 ± 2.0	22.6 ± 2.0	40.8 ± 2.0
**1.5 (low PEF)**	48.6 * ± 1.0	28.5 * ± 1.0	47.9 * ± 1.0
**3.0 (high PEF)**	42.7 ± 2.0	24.8 * ± 2.0	43.4 ± 4.0

* Represents Dunnett’s testing values that are significant (*p* < 0.05).

## Data Availability

The original contributions presented in the study are included in the article, further inquiries can be directed to the corresponding author.
